# Changes on computed tomography in post-acute COVID-19 syndrome: systematic review and meta-analysis

**DOI:** 10.1590/0100-3984.2025.0012-en

**Published:** 2025-11-13

**Authors:** Tatiane Peroba Araújo, Glécia Virgolino da Silva Luz, Marília Miranda Forte Gomes, André Luís Sousa Araújo, Welington Silva

**Affiliations:** 1 Secretaria de Saúde do Distrito Federal, Brasília, DF, Brazil; 2 Programa de Pós-graduação em Engenharia Biomédica (PPGEB), Universidade de Brasília, Brasília, DF, Brazil; 3 Centro Universitário do Planalto Central Aparecido dos Santos (UNICEPLAC), Brasília, DF, Brazil; 4 Ministério da Saúde do Brasil, Brasília, DF, Brazil; 5 Procuradoria Geral da União, Brasília, DF, Brazil.; 6 Serviço Federal de Processamento de Dados (SERPRO), Brasília, DF, Brazil

**Keywords:** COVID-19, Tomography, X-ray computed, Post-acute COVID-19 syndrome, Thorax, Lung diseases/complications., COVID-19, Tomografia computadorizada, Síndrome pós-COVID-19, Tórax, Doença pulmonar crônica.

## Abstract

The objective of this systematic review and meta-analysis of observational studies was to estimate the prevalence of residual alterations in the lung parenchyma on computed tomography (CT) after coronavirus disease 2019 (COVID-19), correlating those alterations with the severity of the acute phase of the disease. We reviewed data related to adult patients evaluated at 3, 6, and 12 months after the diagnosis of moderate-to-critical COVID-19. We performed structured searches of 14 databases, encompassing works published between January 2020 and January 2024. Thus, 44 primary studies were selected. Data on mild cases of COVID-19 were excluded, as were those related to assessment of the acute phase of the disease. The results were analyzed descriptively, and meta-analyses were conducted to estimate prevalence. The estimated prevalence of altered CT scans at post-diagnosis months 3, 6, and 12 was 69.0% (95% CI: 60.0-77.6%; *I*^2^ = 86%; *p* < 0.001), 62.0% (95% CI: 52.0-71.5%; *I*^2^ = 77%; *p* < 0.001), and 54.0% (95% CI: 40.0-67.5%; *I*^2^ = 88%; *p* < 0.001), respectively. There was no correlation between severity of the acute phase and the persistence of alterations on CT in general. Among the CT scans acquired at post-diagnosis month 3, alterations indicative of fibrosis were observed in 22% (95% CI: 13-30%; *I*^2^ = 85%; *p* < 0.001), and no reduction in that prevalence was observed at the subsequent time points (rho-s = 0.952; *p* < 0.000). The severity of the acute phase showed a positive correlation with the presence of lesions indicative of pulmonary fibrosis on CT scans acquired at 3 months after the diagnosis of COVID-19.

## Abstract (pt)

Trata-se de uma revisão sistemática com metanálise de estudos observacionais, com o objetivo de estimar a prevalência de alterações tomográficas residuais no parênquima pulmonar, em correlação com a gravidade da doença aguda. Foram observados os marcos temporais 3, 6 e 12 meses após o diagnóstico de COVID-19 moderada a crítica em adultos. Foi feita busca estruturada em 14 bases de dados, englobando trabalhos publicados de janeiro/2020 a janeiro/2024, selecionando-se 44 estudos primários. Dados sobre casos leves de COVID-19 e avaliação da fase aguda da doença foram excluídos. Os resultados foram analisados descritivamente e metanálises foram conduzidas para derivar estimativas de prevalência. A prevalência estimada de tomografias alteradas foi 69% (IC 95%: 60-77,6%; *I*^2^ = 86%; *p* < 0,001) aos 3 meses; 62% (IC 95%: 52-71,5%; *I*^2^ = 77%; *p* < 0,001) aos 6 meses; e 54% (IC 95%: 40-67,5%; *I*^2^ = 88%; *p* < 0,001) aos 12 meses. Não houve correlação entre gravidade da fase aguda e persistência de lesões tomográficas gerais. Em 22% (IC 95%: 13-30%; *I*^2^ = 85%; *p* < 0,001) das tomografias de 3 meses foram observados indicativos de fibrose, sem redução da prevalência nos controles subsequentes (ρs = 0,952; *p* < 0,000). A gravidade da fase aguda apresentou correlação positiva com a presença de lesões tomográficas indicativas de fibrose pulmonar aos 3 meses após COVID-19.

## INTRODUCTION

Post-acute coronavirus disease 2019 (post-acute COVID-19) syndrome is characterized by new or persistent symptoms, unexplained by another diagnosis, for more than 12 weeks after an infection consistent with COVID-19^**(1)**^. Although the extent of lung lesions in the acute phase is considered a risk factor, mild acute symptoms can also evolve to post-acute COVID-19 syndrome^**(2)**^. The progression of the disease has been monitored in various studies. One example is the multicenter COVID-FIBROTIC cohort study^**(3)**^, whose participants presented with a range of imaging characteristics on computed tomography (CT) at one year after diagnosis with COVID-19, including complete resolution (in 63.0%) and chronic fibrotic changes (in 29.4%). The distinction between a slowly resolving inflammatory process and potentially irreversible lesions can be made by analyzing the temporal behavior of lung lesions on chest CT.

In the context outlined above and in the interest of evidence-based health care, a systematic review can synthesize the knowledge currently available in the scientific literature. Such a study design combines the results of various primary studies, increasing the sample size and the statistical power of the results. Therefore, to estimate the prevalence of residual CT alterations in the lung parenchyma, in correlation with the severity of the acute phase of the disease, we conducted a systematic review and meta-analysis of observational studies. We synthesized data related to adult patients evaluated at 3, 6, and 12 months after the diagnosis of moderate-to-severe COVID-19.

## METHOD

This systematic review was conducted in accordance with the Preferred Reporting Items for Systematic Review and Meta-Analysis (PRISMA) guidelines and was registered with the International Prospective Register of Systematic Reviews^**(4)**^ (Registration no. CRD42024572100).

The study question was formulated by using the Population Exposure Comparator Outcome framework, through which we defined the population as adults; the exposure as a confirmed case of moderate to critical acute COVID-19 requiring hospitalization, at least 3 months prior to the CT scan; the comparator as a normal chest CT scan; and the outcome as residual lung changes. The search strategy involved the following keywords (based on the Medical Subject Headings and Excerpta Medica Tree): “adult”; “SARS-CoV-2”; “COVID-19”; “long COVID”; “tomography”; “pulmonary fibrosis”; and “chronic interstitial lung disease”. In addition to the inclusion criteria described in the Population-Exposure-Comparator-Outcome framework, the selection of full texts included randomized or non-randomized observational studies, either cohort studies or case-control studies. Studies evaluating mild cases of COVID-19 were excluded, as were those assessing only the acute phase of the disease. The characteristics of the CT findings were described in accordance with the Fleischner Society glossary of terms for CT imaging. Statistical analysis was performed with the Statistical Package for the Social Sciences, version 3.0 (SPSS Inc., Chicago, IL, USA).

A structured search was conducted between 17 January and 20 January, 2024, in 14 databases, including research protocols, unpublished data sources, and gray literature: Embase; Brazilian Virtual Library of Health (Latin-American and Caribbean Health Sciences Literature); Medline/PubMed; Scopus (Elsevier); Web of Science; Cochrane and the Cochrane Database for COVID-19 Studies; ProQuest Dissertations; International Standard Randomised Controlled Trial Number Registry; ClinicalTrials.gov; Brazilian Registry of Clinical Trials; European Union Clinical Trial Register; University of London; and Google Scholar. There were no restrictions on the language of publication. Studies published from January 2020 through January 2024 were included. Two of the authors, working independently, identified eligible texts. In case of disagreement, a third author was consulted. The data were extracted into standardized tables. We extracted data on the characteristics of the primary studies and their study populations, as well as on the lung alterations described on chest CT scans and the use of artificial intelligence (AI) in the CT assessment process. The primary outcome measure was the prevalence of CT alterations in the lung parenchyma at 3, 6, and 12 months after the diagnosis of COVID-19. Those time points were defined based on those most commonly evaluated in the primary studies included. In addition, they allow observation of the radiological evolution in distinct phases of recovery: early convalescence (3 months); the intermediate phase (6 months); and the late/potential sequelae phase (12 months).

Categorical variables are reported as absolute values and percentages, whereas continuous variables are reported as means and standard deviations. Summary measures for the pooled prevalence estimates of the primary outcome measure were calculated by meta-analysis under a random-effects model and are presented as forest plots. The certainty level for inferences was 95%. Comparisons between the mean prevalence of each CT alteration at each time point were made with the paired-samples t-test (t) for variables with normal data distribution or with the Wilcoxon signed-rank test (Z) for those with nonparametric distribution. Spearman’s correlation coefficient (rho-*s*) was calculated to assess the correlation between the prevalence of a given CT alteration and the severity of the acute phase of COVID-19, the correlation being considered significant at the 0.05 level, for both ends. Statistical heterogeneity was assessed by using the Higgins test to calculate the *I*^2^ statistic, on the basis of which, the heterogeneity was classified as low (*I*^2^ < 50%) or high (*I*^2^ > 50%), and a qualitative analysis was performed to determine the dispersion of the sample data. Subgroup analyses were performed according to the outcomes and levels of severity evaluated. The methodological quality of primary studies was assessed by using the Newcastle-Ottawa scale for observational studies, and publication bias was analyzed by using funnel plots and Egger’s test.

## RESULTS

### General characteristics of primary studies

**Figure 1:**
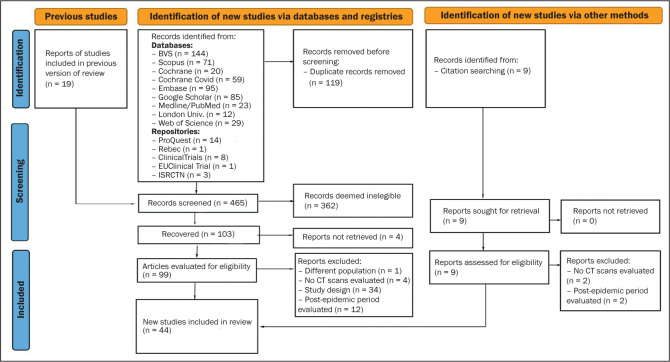
PRISMA flow chart.

**Figure 2:**
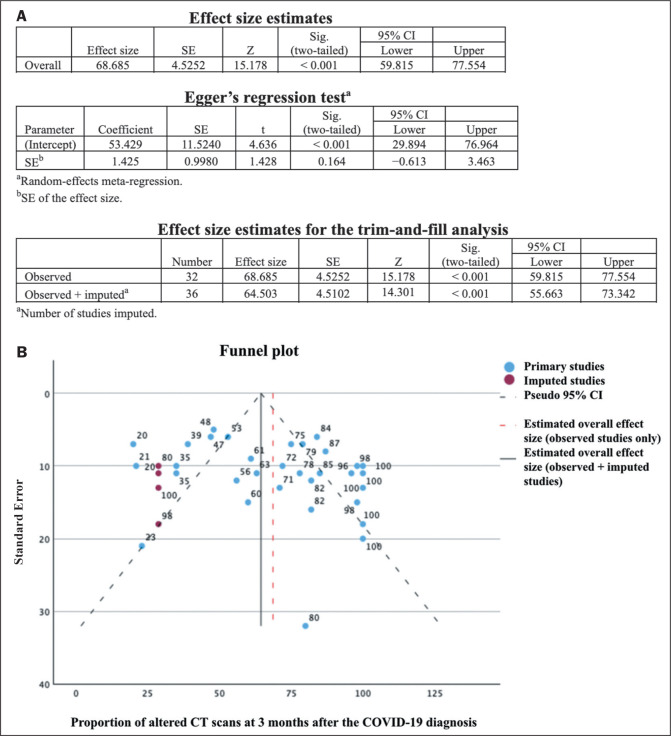
Analysis of publication bias for primary studies that evaluated residual lung changes on chest CT at 3 months after the diagnosis of COVID-19. Statistical tests (A) and funnel plot (B).

A total of 565 articles were retrieved via the search strategy. From among those, 44 primary studies were selected (Figure 1), including cohort studies (n = 38) and case-control studies (n = 6). Of those 44 studies, 31 were prospective and 13 were retrospective. The methodological quality was considered good, with a score of 7 or 8 on the Newcastle-Ottawa scale, in 34 of the studies and fair in the others^**(5-14)**^. No significant publication bias was observed in the sample for the various outcomes (Egger *p* > 0.05), as illustrated in Figure 2. The countries with the highest numbers of articles were China (n = 12) and Italy (n = 9). In three studies, a low-dose radiation protocol was employed for chest CT (Table 1). Nine studies used AI to aid in the identification and quantification of CT lesions. Table 2 lists the AI-based programs used and correlates them with the countries where the studies were conducted. The various types of image interpretation software were designed to identify normal lung parenchyma or to recognize diseases in the parenchyma^**(15,16)**^.

**Table 1:** General characteristics of primary studies of post-acute COVID-19 syndrome.

Study	Year	Setting	Study design	Timeline	Acute phase severity	Country	Revista	Al	Low-dose CT
Baratella et al.^(5)^	2021	Single-center	Case-control	Retrospective	Severe	Italy	JCM	No	No
Bardakci et al.^(42)^	2021	Single-center	Cohort	Prospective	Severe	Turkey	JMV	No	No
Bernardinello et al.^(17)^	2023	Single-center	Cohort	Prospective	Severe	Italy	Front Med	No	No
Besutti et al.^(43)^	2022	Multicenter	Cohort	Retrospective	Severe	Italy	Tomography	No	No
Bocchino et al.^(18)^	2022	Single-center	Cohort	Prospective	Moderate	Italy	Radiology	No	No
Caruso et al.^(44)^	2021	Single-center	Cohort	Prospective	Moderate	Italy	Radiology	Yes	No
Chen et al.^(6)^	2021	Single-center	Cohort	Prospective	Severe	China	BMCM	No	No
Eberst et al.^(20)^	2022	Single-center	Cohort	Prospective	Severe	France	AIC	No	No
CIBERESUCIC0VID^(21)^	2022	Multi center	Cohort	Prospective	Severe	Spain	Front Med	No	No
COVID-BioB Study^(40)^	2022	Single-center	Cohort	Prospective	Severe	Italy	JCVA	Yes	No
Mulet et al.^(3)^	2023	Single-center	Cohort	Prospective	Severe	Spain	AJRCMB	No	No
Luger et al.^(19)^	2022	Single-center	Cohort	Prospective	Moderate/Severe	Austria	Radiology	Yes	Yes
PHENOTYPE Study^(38)^	2022	Single-center	Cohort	Prospective	Moderate/Severe	United Kingdom	Radiology	No	No
Stewart et al.^(12)^	2023	Multi center	Cohort	Prospective	Severe	United Kingdom	AJRCCM	No	No
Farghaly et al.^(7)^	2022	Single-center	Case-control	Retrospective	Severe	Saudi Arabia	Medicine	No	No
Faverio et al.^(46)^	2022	Multicenter	Cohort	Prospective	Moderate	Italy	RR	No	No
Froidure et al.^(22)^	2021	Single-center	Cohort	Prospective	Severe	Belgium	RM	Yes	No
Han et al.^(8)^	2021	Multi center	Case-control	Prospective	Severe	China	Radiology	No	No
Huang et al.^(9)^	2023	Single-center	Case-control	Retrospective	Moderate	China	Front Med	Yes	No
Karampitsakos et al.^(23)^	2023	Multi center	Cohort	Retrospective	Moderate	Greece	Front Med	Yes	No
Kumar et al.^(24)^	2023	Single-center	Cohort	Retrospective	Moderate/Severe	United Kingdom	Clin Med	No	No
Kurys-Denis et al.^(25)^	2022	Single-center	Cohort	Retrospective	Moderate/Severe	Poland	PAIM	No	No
Lazar et al.^(26)^	2022	Single-center	Case-control	Prospective	Severe	Romania	Diagnostics	Yes	No
Li et al.^(11)^	2021	Single-center	Cohort	Prospective	Severe	China	RR	No	No
Liao et al.^(47)^	2021	Single-center	Cohort	Prospective	Severe	China	IDT	Yes	No
Huang et al.^(15)^	2021	Single-center	Cohort	Prospective	Severe	China	The Lancet	No	No
Lorent et al.^(27)^	2022	Multi center	Cohort	Prospective	Moderate/Severe	Belgium	ERJOR	No	No
Marando et al.^(39)^	2022	Single-center	Cohort	Prospective	Severe	Switzerland	npjPCRM	No	Yes
Mostafa et al.^(28)^	2023	Single-center	Cohort	Prospective	Moderate/Severe	Egypt	EJCDT	No	No
Nabahati et al.^(29)^	2021	Single-center	Cohort	Prospective	Moderate	Iran	EJRNM	No	No
Pan et al.^(37)^	2022	Multicenter	Cohort	Prospective	Severe	Canada	Radiology	No	No
Poerio et al.^(30)^	2022	Single-center	Cohort	Retrospective	Moderate/Severe	Italy	SNCCM	No	No
Polat et al.^(31)^	2022	Single-center	Case-control	Retrospective	Moderate/Severe	Turkey	RC	No	No
Russo et al.^(48)^	2022	Single-center	Cohort	Prospective	Severe	Italy	IEM	No	No
Lenoir et al.^(10)^	2023	Multicenter	Cohort	Prospective	Grave	Switzerland	Respiration	No	No
van Raaij et al.^(32)^	2022	Single-center	Cohort	Prospective	Moderate/Severe	Netherlands	RMR	No	No
Vural et al.^(33)^	2021	Single-center	Cohort	Retrospective	Severe	Turkey	Tuberk Toraks	No	Yes
Wu etal.^(36)^	2021	Single-center	Cohort	Prospective	Severe	China	The Lancet	No	No
Li et al.^(49)^	2021	Multi center	Cohort	Prospective	Moderate/Severe	China	EJR	No	No
Zhang et al.^(13)^	2021	Single-center	Cohort	Retrospective	Severe	China	ER	No	No
Zhao et al.^(34)^	2020	Multicenter	Cohort	Retrospective	Moderate	China	ECM	No	No
Zhou et al.^(35)^	2021	Multicenter	Cohort	Prospective	Severe	China	Front Med	Yes	No
Zou et al.^(16)^	2021	Single-center	Cohort	Prospective	Severe	China	PLoS One	Yes	No
Zubairi et al.^(14)^	2021	Single-center	Cohort	Retrospective	Severe	Pakistan	ARM	No	No

JCM, Journal of Clinical Medicine; JMV, Journal of Medical Virology; Front Med, Frontiers in Medicine; BMCM, BMC Medicine; AIC, Annals of Intensive Care; JCVA, Journal of Cadiothoracic and Vascular Anesthesia; AJRCMB, American Journal of Respiratory Cell and Molecular Biology; AJRCCM, American Journal of Respiratory and Critical Care Medicine; RR, Respiratory Research; RM, Respiratory Medicine; Clin Med, Clinical Medicine; PAIM, Polish Archives of Internal Medicine; IDT, Infectious Diseases and Therapy; ERJOR, ERJ Open Research; npjPCRM, npj Primary Care Respiratory Medicine; EJCDT: The Egyptian Journal of Chest Diseases and Tuberculosis; EJRNM, Egyptian Journal of Radiology and Nuclear Medicine; SNCCM, SN Comprehensive Clinical Medicine; RC, Respiratory Care; IEM, Internal and Emergency Medicine; RMR, Respiratory Medicine and Research; EJR, European Journal of Radiology; ER, European Radiology; ECM, EClinicalMedicine; ARM, Advances in Respiratory Medicine.

**Table 2:** Use of AI in primary studies of post-acute COVID-19 syndrome.

Software/method	Study - country, year
Quantitative CT Assessment System for COVID-19 (YT-CT-Lung, YITU Healthcare Technology Co., Ltd, Hangzhou,	Zhou et al.^(35)^- China, 2021
China)	Zou etal.^(16)^-China, 2021
Imbio Lung Texture Analysis (Imbio LLC, Minneapolis, MN, USA)	Karampitsakos et al.^(23)^ - Greece, 2023
Syngo.via CT Pneumonia Analysis (Siemens Healthineers, Erlangen, Germany)	Luger et al.^(19)^ - Austria, 2022 Froidure et al.^(22)^ - Belgium, 2021 Lazar et al.^(26)^ - Romania, 2022
Thoracic VCAR (GE Healthcare, Chicago, IL, USA)	Caruso et al.^(44)^ - Italy, 2021
Intellispace (Philips Medical Systems, Best, The Netherlands)	COVID-BioB Study^(40)^- Italy, 2022
Deep lung parenchyma enhancing (computer-aided detection) method	Huang et al.^(9)^ - China, 2023

**Figure 3:**
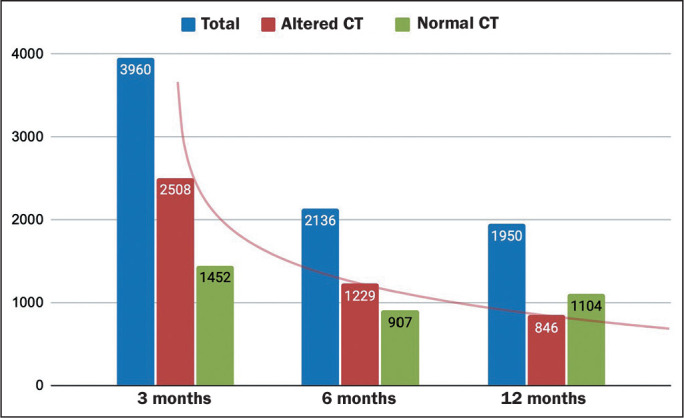
Assessment of lung parenchyma by computed CT: overall count of examinations (normal and altered).

**Figure 4:**
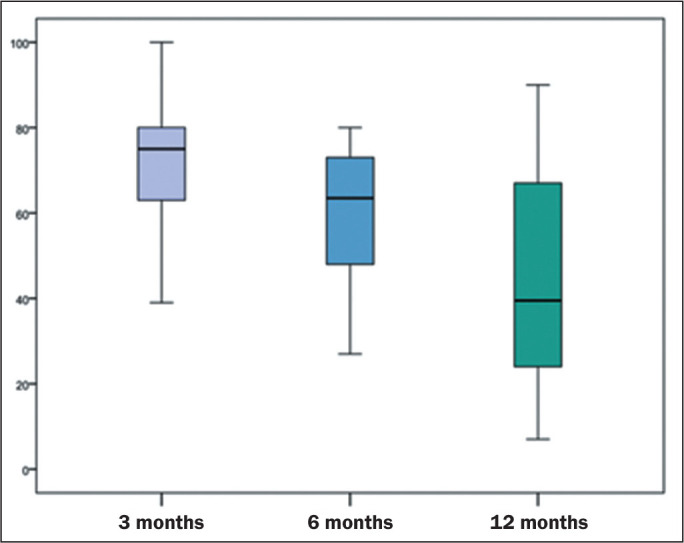
Distribution of the relative frequencies of altered CT scans in the primary studies, regardless of the type of alteration found.

Among the 44 studies selected, 32 (72.7%), 19 (43.2%), and 21 (47.7%), respectively, reported CT findings obtained at 3 months^**(5,6,10-14,16-40)**^, 6 months^**(3,6,7,9,11,15,18,19,23,29,31,32,36,37,41-45)**^, and 12 months^**(6-8,10,11,15,17-20,24,27,32,36-41,43,46)**^ after the COVID-19 diagnosis. Six studies (13.6%) reported CT findings obtained at all three time points^**(6,18,19,36,37,41)**^. Collectively, the 44 studies evaluated 8,046 CT scans acquired in 5,776 participants, given that some participants were evaluated at more than one time point. Of those 5,776 participants, 59% of were men and 41% were women; the mean age was 57 ± 8 years; the acute phase of COVID-19 was classified as severe/critical in 71% of the participants and as moderate in 29%. Figure 3 illustrates the numbers of CT examinations categorized by time point and finding. Figure 4 shows the distribution of the relative frequencies of altered CT scans.

### Combined CT findings: meta-analysis

**Figure 5:**
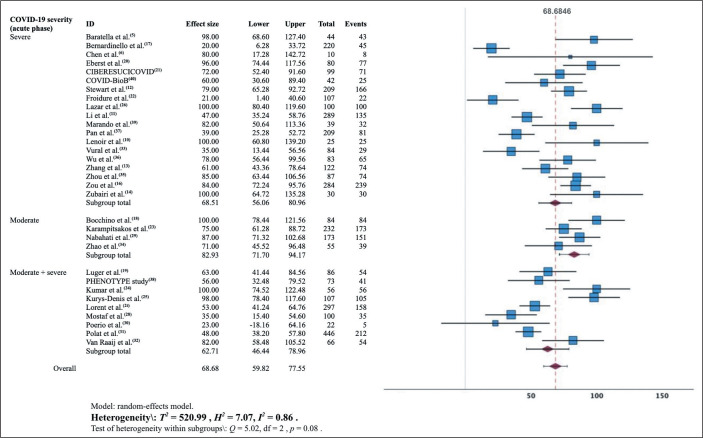
Forest plot showing the estimated prevalence of residual lesions in the lung parenchyma at 3 months after the diagnosis of COVID-19.

**Figure 6:**
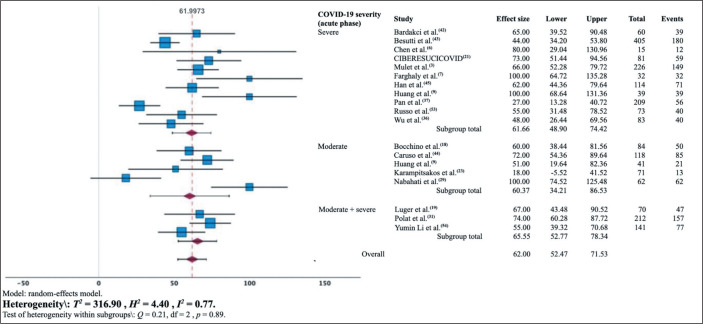
Forest plot showing the estimated prevalence of residual lesions in the lung parenchyma at 6 months after the diagnosis of COVID-19.

**Figure 7:**
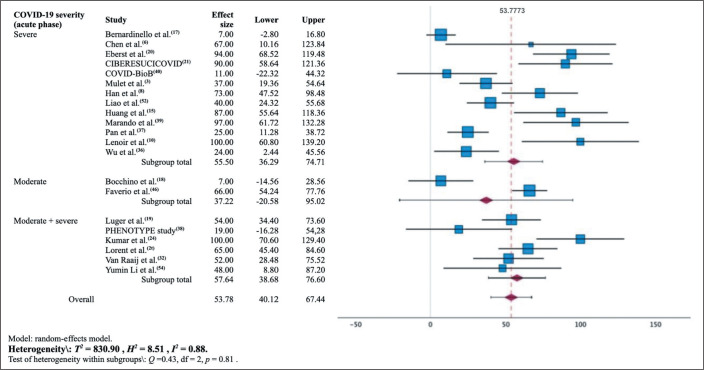
Forest plot showing the estimated prevalence of residual lesions in the lung parenchyma at 12 months after the diagnosis of COVID-19.

The prevalence of residual lesions in the lung parenchyma was defined as the ratio between the number of CT scans showing any alteration and the total number of CT scans available in the period defined. The results of the meta-analyses are detailed in the forest plots displayed in Figures 5, 6, and 7.

The pooled prevalence of residual lesions on 3-month CT scans estimated in the meta-analysis was 69% (95% CI: 60.0-77.6%; *I*^2^ = 86%; *p* < 0.001). That meta-analysis included data from 32 studies involving 3,960 participants. In that sample, the prevalence ranged from 20%^**(17)**^ to 100%^**(10,14,18,26,38)**^. The meta-analysis stratified by the severity of the acute phase of COVID-19 resulted in an estimated pooled prevalence of 68.5% (95% CI: 56.0-81.0%) in the group in which it was classified as severe and 83% (95% CI: 72-94%) in the group in which it was classified as moderate.

The pooled prevalence of at least one lesion seen on the 6-month CT scan, estimated in the meta-analysis, was 62.0% (95% CI: 52.0-71.5%; *I*^2^ = 77%; *p* < 0.001). In that sample, the prevalence ranged from 18%^**(23)**^ to 80%^**(6)**^. That meta-analysis included data from 19 studies involving 2,136 participants. The meta-analysis stratified by the severity of the acute phase of COVID-19 resulted in an estimated pooled prevalence of 62.0% (95% CI: 49.0-74.0%) in the group in which it was classified as severe and 60.0% (95% CI: 34.0-86.5%) in the group in which it was classified as moderate.

The pooled prevalence of at least one lesion seen on the 12-month CT scan, estimated in the meta-analysis, was 54.0% (95% CI: 40.0-67.5%; *I*^2^ = 88%; *p* < 0.001). In that sample, the prevalence ranged from 7%^**(17,18)**^ to 80%^**(39)**^. That meta-analysis included data from 21 studies involving 1,950 participants. The meta-analysis stratified by the severity of the acute phase of COVID-19 resulted in an estimated pooled prevalence of 55.5% (95% CI: 36.0-75.0%) in the group in which it was classified as severe and of 37.0% (95% CI: 0.0-95.0%) in the group in which it was classified as moderate.

The rate of reduction in the prevalence of altered CT scans was calculated in order to assess the temporal evolution over a one-year period after the diagnosis of COVID-19 (Table 3). There was a statistically significant reduction in the mean prevalence of residual lesions on CT scans between post-diagnosis months 3 and 12, as well as between post-diagnosis months 6 and 12, with reductions of 18% (Z = -1,922; *p* = 0.027) and 13% (t(9) = 1,990; *p* = 0.039), respectively. There was no significant reduction in the prevalence of such lesions between post-diagnosis months 3 and 6 (Z = 1.26; *p* = 0.208).

**Table 3:** Statistical (one-tailed) comparison between time points for paired samples in terms of the prevalence of residual lung lesions on chest CT: temporal evolution over one year after the diagnosis of COVID-19.

CT finding	P^*^	P†	A
Any alteration (month 3^*^ vs. month 6)	0.205	0.136	↓ 10.5%
Any alteration (month 3^*^ vs. month 12)	0.027	0.019	↓ 17.9%
Any alteration (month 6 vs. month 12)	0.037	0.039	↓ 12.6%
Fibrosis^8^ (month 3^*^ vs. month 6)	0.104	0.257	↑2.9%
Fibrosis^8^ (month 3^*^ vs. month 12)	0.120	0.195	↑4.6%
Fibrosis^8^ (month 6^*^ vs. month 12^*^)	0.118	0.120	↑7.6%

* Wilcoxon signed-rank test.

† Paired t-test. ∆, mean difference between the values.

‡ Samples with non-normal distribution.

§ Lesions associated with pulmonary fibrosis (honeycombing or traction bronchiectasis/bronchiolectasis).

### CT findings suggestive of fibrosis: meta-analysis

**Figure 8:**
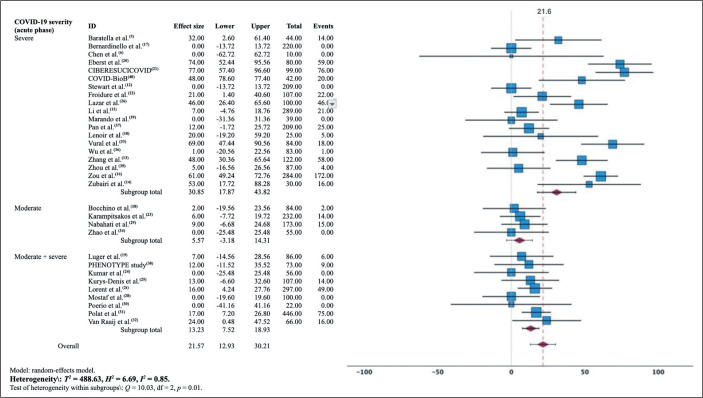
Estimated prevalence of pulmonary changes related to pulmonary fibrosis at three months after the diagnosis of COVID-19, categorized by severity during the acute phase of the disease.

Various CT findings were classified by primary studies as lesions related to pulmonary fibrosis. The most commonly described findings were honeycombing and traction bronchiectasis. A meta-analysis of the prevalence of this subgroup demonstrated wide data dispersion (Figure 8; Table 3). For this review, the presence of honeycombing or traction bronchiectasis was considered indicative of fibrosis.

The estimated pooled prevalence of pulmonary fibrosis at 3, 6, and 12 months after the COVID-19 diagnosis was 22% (95% CI: 13-30%; *I*^2^ = 85%; *p* < 0.001), 23% (95% CI: 10-35; *I*^2^ = 87%; *p* < 0.001), and 24% (95% CI: 10-37.5%; *I*^2^ = 88%; *p* < 0.001), respectively. There was no significant reduction in fibrosis prevalence between post-diagnosis months 3 and 12 (*p* = 0.120) or between post-diagnosis months 6 and 12 (*p* = 0.118). The Wilcoxon signed-rank test corroborated the hypothesis that fibrotic lesions seen on CT scans acquired at month 3 persisted at month 6 (Z = 1.26; *p* = 0.208) and at month 12 (Z = 1.17; *p* = 0.241), as detailed in Table 3. For the 3-month time point, the meta-analysis stratified by severity of the acute phase resulted in a pooled estimate of 31.0% (95% CI: 18.0-44.0%) in the group in which it was classified as severe and of 5.5% (95% CI: 0.0-14.0%) in the group in which it was classified as moderate.

Statistical heterogeneity (expressed as the *I*^2^ statistic), also called inconsistency, is the measure of how different the elements of a study sample are from each other^**(50)**^. In the case of systematic reviews, the *I*^2^ statistic quantifies the differences between the primary studies included in the review, because they are the units of occurrence of the sample. However, the intrinsic diversity of the population of each primary study indirectly affects interpretation of the results of the review^**(51)**^. The forest plots presented in this work (Figures 5 to 8) summarize, individually and in combination, the effect estimate (prevalence of altered CT scans) and the confidence intervals (dispersion). There was considerable heterogeneity among the studies in our sample in terms of the prevalence of altered CT scans, even when stratified by the severity of the acute phase of COVID-19. That could limit the representativeness of the results in relation to the general population, given that other characteristics of the participants may have contributed to the inconsistency.

### Correlation between variables

**Figure 9:**
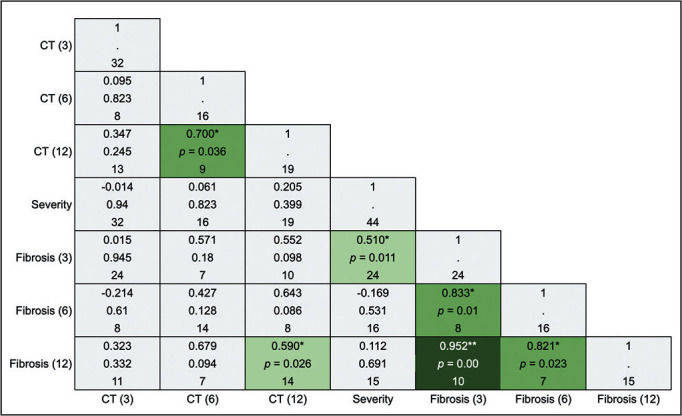
Spearman’s correlation coefficients for the comparison between the severity of the acute phase of COVID-19 and the presence of changes on chest CT (for all alterations and for only those indicative of fibrosis) at 3, 6, and 12 months after the diagnosis of COVID-19-(3), (6), and (12), respectively.

The correlations that the severity of the acute phase of COVID-19 showed with the presence of altered CT scans at the three time points and with specific alterations are illustrated in Figure 9. The acute phase of COVID-19 being classified as severe or critical showed a moderate correlation with the presence of lesions indicative of fibrosis on the 3-month CT scan (rho-*s =* 0.510; *p* = 0.011). The presence of fibrotic alterations on the 3-month CT scan showed strong and very strong positive correlations, respectively, with their persistence on the 6-month CT scan (rho-*s =* 0.833; *p* = 0.01) and on the 12-month CT scan (rho-*s =* 0.952; *p* < 0.001). No correlation was observed between the severity of the acute phase and the overall presence of lesions on the 3-month CT scan (rho-*s* = -0.014; *p* = 0.94), 6-month CT scan (rho-*s* = 0.061; *p* = 0.823) or 12-month CT scan (rho-*s* = 0.205; *p* = 0.399).

### Specific CT findings

**Figure 10:**
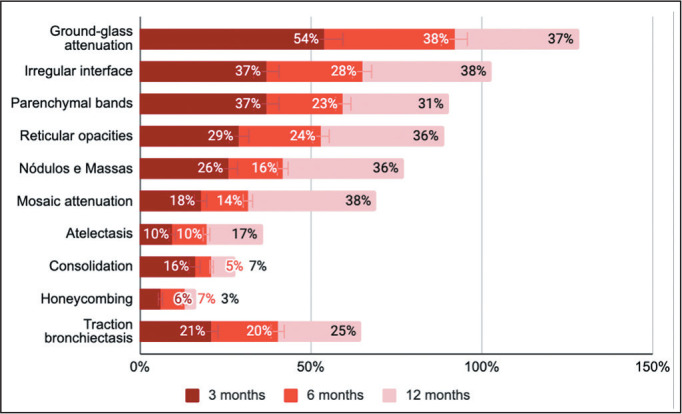
Mean relative frequencies of changes on chest CT scans described in primary studies, categorized by observation period (n = 44 studies).

The mean relative frequencies of the main CT alterations reported in the primary studies are shown in Figure 10 and Table 4. The proportions were calculated in relation to the total number of examinations performed during the study period.

**Table 4:** Relative frequency of lung changes detected on chest CT at 3, 6, and 12 months after the diagnosis of COVID-19.

Finding	Mean ± SD	Median (IQR)	n	Distribution	Range	*P*
Parenchymal consolidation, %						
At month 3	16 ± 3.4	9 (3-20.3)	18	Normal	1-70	< 0.001
At month 6	5 ± 1.8	3.5 (1-8.3)	8	Non-normal	1-15	0.065
At month 12	6.6 ± 1.5	5 (2-10)	13	Non-normal	1-20	0.084
Ground glass attenuation, %						
At month 3	53.7 ±4.2	57 (37-73)	31	Normal	8-94	0.499
At month 6	37.9 ± 5.8	36.5 (20-47.5)	16	Non-normal	7-91	0.071
At month 12	36.5 ± 5.4	33 (14-52.5)	20	Non-normal	2-84	0.391
Atelectasis, %						
At month 3	9.5 ±4.5	7 (2.5-19)	4	Non-normal	2-22	0.404
At month 6	10 ± 2.8	10 (3-15.8)	6	Non-normal	3-93	0.577
At month 12	16.5 ± 8.2	15 (2.3-32.3)	4	Non-normal	2-34	0.180
Parenchymal bands, %						
At month 3	37 ± 5.1	34 (18-45)	19	Non-normal	7-84	0.150
At month 6	22.5 ±3.5	24 (8.5-32)	13	Non-normal	3-44	0.446
At month 12	31 ± 7.2	25 (4-45)	15	Non-normal	1-84	0.052
Reticulation (septal thickening or interstitial irregularities), %						
At month 3	28.5 ±4.1	25 (11-40)	27	Normal	2-86	0.011
At month 6	24.3 ± 5	19 (7-37)	17	Non-normal	2-77	0.064
At month 12	36 ± 6.4	36 (15.5-48)	17	Non-normal	1-100	0.274
Traction bronchiectasis or bronchiolectasis, %						
At month 3	20.8 ± 4.1	13(7-13)	23	Normal	1-77	< 0.001
At month 6	19.5 ± 5.5	13 (10-24.5)	13	Normal	1-26	< 0.001
At month 12	24.6 ± 6.5	15.5 (5-37.3)	16	Normal	1-69	0.011
Traction bronchiectasis or bronchiolectasis (no outliers), %						
At month 3	19 ± 3.4	13(7-29.5)	22	Normal	2-66	0.004
At month 6	14.5 ± 2.4	12.5 (9.5-23.3)	11	Non-normal	1-26	0.472
At month 12	20.3 ±5.1	11 (5-35)	15	Normal	1-69	0.036
Honeycombing						
At month 3	16.6 ± 4.2	10 (4.5-33.8)	12	Normal	1-40	0.039
At month 6	19.8 ± 8.5	12 (2.3-29.5)	8	Normal	1-72	0.027
At month 12	21.6 ± 10.4	11 (3-45.5)	5	Non-normal	1-56	0.318
Irregular lung interface, %						
At month 3	36.8 ± 9.3	25 (12.5-66.5)	9	Non-normal	4-84	0.242
At month 6	27.9 ± 6.4	26 (10-45.3)	8	Non-normal	8-52	0.157
At month 12	38 ± 12.1	36(6-73)	7	Non-normal	6-76	0.078
Nodules or masses, %						
At month 3	26 ± 7	26 (19-33)	2	-	19-33	-
At month 6	15.8 ± 8	23.5 (4.5-29.5)	5	Non-normal	0-47	0.057
At month 12	35.5 ± 8.5	35.5 (27-35.5)	2	-	27-44	-
Mosaic attenuation, %						
At month 3	17.5 ± 10.2	6(3-19.5)	9	Normal	1-96	< 0.001
At month 6	13.8 ± 9.1	5.5 (3.3-32.5)	4	Normal	3-41	0.015
At month 12	37.7 ± 15	23 (4-90)	7	Normal	2-96	0.049

%, in relation to the total number of examinations performed at the time point; n, number of studies that performed the test; IQR, interquartile range.

Of the 3,981 CT scans performed at 3 months after the COVID-19 diagnosis, 2,508 (63.0%) were altered. The most commonly observed alterations were ground-glass attenuation (in 54.0 ± 4.2%), parenchymal bands (in 37.0 ± 5.1%), reticular opacities (in 29.0 ± 4.1%), nodules/masses (in 26.0 ± 7.0%), traction bronchiectasis (in 21.0 ± 4.1%), and mosaic attenuation (in 18.0 ± 10.0%).

Of the 2,137 CT scans performed at 6 months after the COVID-19 diagnosis, 1,229 (57.5%) were altered. The most commonly observed lesions were ground-glass attenuation (in 38.0 ± 5.8%), reticular opacities (in 24.0 ± 5.0%), parenchymal bands (in 23.0 ± 3.5%), traction bronchiectasis (in 20.0 ± 5.5%), nodules/masses (in 16.0 ± 8.0%), and mosaic attenuation (in 14.0 ± 1.8%).

Of the 1,967 CT scans performed at 12 months after the COVID-19 diagnosis, 846 (43.0%) were altered. The most commonly observed lesions were mosaic attenuation (in 38.0 ± 15.0%), ground-glass attenuation (in 37.0 ± 5.4%), nodules/masses (in 36.0 ± 8.5%), reticular opacities (in 36.0 ± 6.4%), parenchymal bands (in 31.0 ± 7.2%), and traction bronchiectasis (in 25.0 ± 6.5%).

Meta-analyses were generated to estimate the pooled prevalence for each type of lesion seen on CT at each of the three time points evaluated^**(4)**^. Those results were compared with each other to analyze their temporal behavior (Table 5). Over the course of the observation period, there were statistically significant reductions in ground-glass attenuation, the mean prevalence of which decreased by 22% from post-diagnosis month 3 to post-diagnosis month 6 (*p* = 0.021) and by 21% from post-diagnosis month 3 to post-diagnosis month 12 (*p* = 0.008). There was no significant difference between month 6 and month 12 in terms of the mean prevalence of ground-glass opacity (*p* = 0.068). There was a 5% increase in the pooled prevalence of traction bronchiectasis from month 3 to month 6 (*p* = 0.018). There was no significant difference between month 6 and month 12 in terms of the pooled prevalence of traction bronchiectasis (*p* = 0.061). These findings suggest stability of the lesions at six months after the acute phase of COVID-19. Interstitial thickening (reticulation) remained stable until post-diagnosis month 6 (Wilcoxon signed-rank, *p* = 0.187), with a statistically significant increase of 8% in its pooled prevalence between post-diagnosis months 6 and 12 (*p* = 0.034). The Wilcoxon signed-rank test and the paired-samples t-test showed that there was no difference between the mean prevalence rates of the other alterations over the course of the observation period.

**Table 5:** Statistical (one-tailed) comparison for paired samples between estimated mean prevalences of specific lung lesions observed on chest CT: temporal evolution over one year after COVID-19.

CT finding	P^*^	P†	A
Ground-glass attenuation (month 3^*^ vs. month 6)	0.021	0.027	↓ 22%
Ground-glass attenuation (month 3^*^ vs. month 12)	0.008	0.012	↓ 20.5%
Ground-glass attenuation (month 6 vs. month 12)	0.068	0.058	↓ 6%
Consolidation (month 3^*^ vs. month 6)	0.200	0.094	↓ 3%
Consolidation (3 month 3^*^ vs. month 12)	0.054	0.216	↓ 1.6%
Consolidation (month 6 vs. month 12)	0.242	0.408	↓1%
Atelectasis (month 3 vs. month 6)	0.500	0.364	↑ 1%
Atelectasis (month 3 vs. month 12)	0.286	0.378	↓ 1.3%
Atelectasis (month 6 vs. month 12)	0.296	0.300	↑4.3%
Parenchymal bands (month 3 vs. month 6)	0.232	0.332	↓ 2.7%
Parenchymal bands (month 3 vs. month 12)	0.444	0.409	↑2.5%
Parenchymal bands (month 6 vs. month 12)	0.472	0.372	↑1.9%
Reticulations (month 3^*^ vs. month 6)	0.187	0.300	↓ 2.1%
Reticulations (month 3^*^ vs. month 12)	0.323	0.408	↓ 1%
Reticulations (month 6 vs. month 12)	0.034	0.043	↓ 8.3%
Traction bronchiectasis (month 3$ vs. month 6^*^)	0.021	0.018	↑5.1%
Traction bronchiectasis (month 3}: vs. month 12^*^)	0.129	0.152	↑3.8%
Traction bronchiectasis (month 6$ vs. month 12^*^)	0.064	0.061	↑6.3%
Honeycombing (month 3^*^ vs. month 6^*^)	0.054	0.094	↑ 6%
Honeycombing (month 3^*^ vs. month 12)	0.054	0.416	↑4%
Honeycombing (month 6^*^ vs. month 12)	0.158	0.094	↑6%
Nodules or masses (month 3 vs. month 6)	0.158	-	↓
Nodules or masses (month 3 vs. month 12)	0.158	-	↑
Nodules or masses (month 6 vs. month 12)	0.327	0.309	↑ 6.5%
Mosaic attenuation (month 3^*^ vs. month 6^*^)	0.090	0.197	↓ 7%
Mosaic attenuation (month 3^*^ vs. month 12)	0.207	0.196	↑15.3%
Mosaic attenuation (month 6^*^ vs. month 12)	0.090	0.205	↑16.7%
Irregular lung interface (month 3 vs. month 6)	0.158	0.035	↓ 4.5%
Irregular lung interface (month 3 vs. month 12)	0.090	0.038	↑ 7%
Irregular lung interface (month 6 vs. month 12)	0.072	0.045	↑14.5%

* Wilcoxon signed-rank test.

† Paired t-test.

‡ Samples with non-normal distribution. ∆, mean difference between the values.

## DISCUSSION

The persistence of symptoms after pneumonia resulting from infection with severe acute respiratory syndrome coronavirus 2 (SARS-CoV-2) is still a relevant issue in medical practice. Some of those symptoms can be related to lung alterations diagnosable by imaging. This systematic review included the sample prevalence of 44 studies on CT alterations in the lungs of adults at 3, 6, and 12 months after moderate-to-severe COVID-19. The pooled prevalence rates were calculated by meta-analysis, and these summary measures were compared by using appropriate statistical tests, not only for analyzing the temporal evolution of lesions but also for correlation with the severity of the acute phase of the disease. The information presented here contributes to the elucidation of the natural history of post-acute COVID-19 syndrome. In addition, it provides a semiotic assessment that assists radiologists in the interpretation of serial examinations, identifying the most common alterations and their propensity for progression.

Approximately 69% of the chest CT scans acquired 3 months after the acute phase of COVID-19 showed some residual lesion. That proportion fell to 62% by month 6 and to 54% by month 12. In comparison with the CT results obtained in month 3, those obtained in month 12 had, on average, returned to normal in 18% of the examinations. Findings related to pulmonary fibrosis were present in 22% of the CT scans acquired at post-diagnosis month 3, and there was no significant reduction in that prevalence from month 3 to month 12. That supports the hypothesis that COVID-19 is associated with pulmonary fibrosis, regardless of the age of the individual^**(52)**^. In this review, we found no correlation between severity in the acute phase of COVID-19 and the persistence of lesions on CT in general. However, there was a moderate correlation between severity in the acute phase and the presence of lesions indicative of pulmonary fibrosis at 3 months after the diagnosis.

Ground-glass opacity was the most common CT alteration at all time points. From post-diagnosis month 3 to post-diagnosis month 6, there was a statistically significant reduction in the prevalence of ground-glass attenuation, as well as a statistically significant increase in the prevalence of traction bronchiectasis, although the number of reticular opacities remained stable. From post-diagnosis month 6 to post-diagnosis month 12, there was no significant change in the prevalence of ground-glass opacity or traction bronchiectasis, suggesting that COVID-19-related lesions stabilize by 6 months after the diagnosis. However, there was a moderately significant increase in the prevalence of reticular opacities between month 6 and month 12.

The meta-analysis revealed high heterogeneity across the different outcomes studied in the post-acute COVID-19 syndrome setting, including general CT findings and fibrotic sequelae. That heterogeneity persisted despite attempts to contain it by stratifying the data by disease severity and specific type of CT finding. The overall prevalence range of pulmonary alterations on chest CT scans was considerably wide for all three of the time points evaluated, especially at post-diagnosis month 3, when it ranged from 20% to 100%.

It is believed that the heterogeneity across studies is attributable, at least in part, to the technique employed to read CT scans, because inconsistencies were observed in the description and classification of lesions, particularly for interstitial changes with irregular interfaces. Future studies could further analyze factors potentially associated with the significant differences between studies, such as age, smoking, and underlying disease. That technique could increase the reliability of the results.

Previous reviews^**(53,54)**^ have also described heterogeneity across studies as a limitation. Bocchino et al.^**(53)**^ conducted a systematic review estimating the prevalence (global and individual) of any type of residual lung alteration related to COVID-19 on chest CT scans acquired at one year after diagnosis, including studies published up through January 2023. The authors estimated the prevalence of such lung alterations at 43.5% (range, 7.1-96.7%), with no influence from the characteristics of interest of the individuals in the study populations (age, sex, smoking history, comorbidities, or severity of the acute phase). They assessed the prevalence of the main lesions seen on CT scans. As in our study, the most common alteration was ground-glass attenuation, with a pooled prevalence of 23.8% and high heterogeneity.

Our study has limitations other than the considerable heterogeneity of the data. We did not have access to previous chest CT scans of the study participants, contributing to a confounding bias between the presence of previous lung disease and lesions resulting from infection with SARS-CoV-2. The lack of detailed clinical data in the studies evaluated in our review precluded a more robust analysis of the correlation between radiological findings and persistent clinical symptoms. In addition, participant COVID-19 vaccination history was not considered. The January 2024 cutoff could be viewed as another limitation. That cutoff was set because the work was conducted within the context of a master’s project with a previously established timeline. However, updating the review would require a new methodological process, given that the PRISMA protocol steps were strictly followed, including double screening, assessment of the risk of bias, and standardized data extraction.

## CONCLUSION

It seems that CT alterations in the lung parenchyma persist in a significant number of patients with post-acute COVID-19 syndrome, even up to 12 months after the acute phase of the disease. Findings associated with pulmonary fibrosis can be observed in approximately 20% of CT scans at the 3-month follow-up evaluation, with no reduction in prevalence in subsequent follow-up examinations. The severity of the acute phase of COVID-19 does not appear to be related to the persistence of lesions on chest CT scans in general. However, severe or critical disease during the acute phase was found to correlate strongly with the presence of lesions indicative of fibrosis on CT scans acquired at 3, 6, and 12 months after the diagnosis of COVID-19. The data indicated an increase in the prevalence of interstitial thickening, with no relevant change in the prevalence of ground-glass attenuation or traction bronchiectasis, after post-diagnosis month 6. The collective sample presented significant heterogeneity, which impedes the generalizability of the results to the general population. Further research could deepen the stratified analysis and elucidate the factors associated with heterogeneity.

## Data Availability

The data supporting the results of this study are published in the body of this article.
